# Bioactive polysaccharides from *Aegle marmelos* fruit: Recent trends on extraction, bio‐techno functionality, and food applications

**DOI:** 10.1002/fsn3.4026

**Published:** 2024-02-20

**Authors:** Madhu Sharma, Aarti Bains, Gulden Goksen, Kandi Sridhar, Minaxi Sharma, Amin Mousavi Khaneghah, Prince Chawla

**Affiliations:** ^1^ Department of Food Technology and Nutrition Lovely Professional University Phagwara Punjab India; ^2^ Department of Microbiology Lovely Professional University Phagwara Punjab India; ^3^ Department of Food Technology, Vocational School of Technical Sciences at Mersin Tarsus Organized Industrial Zone Tarsus University Mersin Turkey; ^4^ Department of Food Technology Karpagam Academy of Higher Education (Deemed to be University) Coimbatore India; ^5^ Department of Applied Biology University of Science and Technology Meghalaya Baridua India; ^6^ Department of Fruit and Vegetable Product Technology Prof. Wacław Dąbrowski Institute of Agricultural and Food Biotechnology, State Research Institute Warsaw Poland

**Keywords:** bael fruit, microwave‐assisted, non‐conventional fruit, polysaccharide extraction

## Abstract

Polysaccharides from non‐conventional sources, such as fruits, have gained significant attention recently. *Aegle marmelos* (Bael), a non‐conventional fruit, is an excellent source of biologically active components with potential indigenous therapeutic and food applications. Apart from polyphenolic components, this is an excellent source of mucilaginous polysaccharides. Polysaccharides are one the major components of bael fruit, having a high amount of galactose and glucuronic acid, which contributes to its potential therapeutic properties. Therefore, this review emphasizes the conventional and emerging techniques of polysaccharide extraction from bael fruit. Insight into the attributes of polysaccharide components, their techno‐functional properties, characterization of bael fruit polysaccharide, emulsifying properties, binding properties, reduction of hazardous dyes, application of polysaccharides in film formation, application of polysaccharide as a nanocomposite, and biological activities of bael fruit polysaccharides are discussed. This review also systematically overviews the relationship between extraction techniques, structural characteristics, and biological activities. Additionally, recommendations, future perspectives, and new valuable insight towards better utilization of bael fruit polysaccharide have been given importance, which can be promoted in the long term.

## Graphical abstract

*Aegle marmelos* is a medium‐sized, slow‐growing subtropical tree and is the only member of the genus *Aegle*. It is an underutilized fruit, an excellent source of biologically active components with potential indigenous therapeutic and food applications. Apart from polyphenolic components, bael is an excellent source of mucilaginous polysaccharides. Therefore, this review emphasizes the conventional and emerging techniques of polysaccharide extraction from bael fruit. Insights into the attributes of polysaccharide components, their techno‐functional properties, characterization of bael fruit polysaccharide, emulsifying properties, binding properties, reduction of hazardous dyes, application of polysaccharides in film formation, application of polysaccharide as a nanocomposite and biological activities of bael fruit polysaccharides are discussed. This review also systematically overviews the relationship between extraction techniques, structural characteristics, and biological activities. Additionally, recommendations, future perspectives, and new valuable insight towards better utilization of bael fruit polysaccharide have been given importance, which can be promoted in the long term.

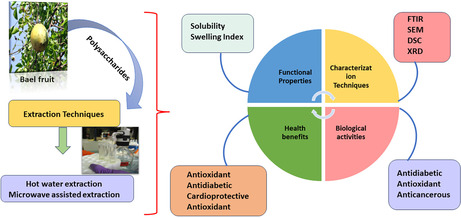

## 1 INTRODUCTION

The demand for safe and healthy food products is increasing globally due to consumer lifestyle changes, urbanization, and technological advancements (Altemimi et al., 2022; Onyeaka et al., 2022; Tripathi et al., 2021). The demand for fruits and vegetables is growing enormously as they contain various vital components, including minerals, vitamins, polyphenols, and polysaccharides, associated with several health benefits (Hou et al., 2019; Nie et al., 2023). Despite having high nutritional and medicinal properties, many fruit plants still need to be utilized/undervalued and discovered (Kaşıkçı & Bağdatlıoğlu, 2022; Şanlıbaba, 2023). Consequently, the bael fruit, a taxonomically classified member of the Rutaceae family, is commonly called the bael fruit. In tropical and subtropical areas, it is the most widely cultivated fruit plant, which is indigenous to Northern India but now found in almost every state of India and also in other countries like Bangladesh, Pakistan, Thailand, Nepal, Burma, Sri Lanka, and China (Bhar et al., 2019; Sharma et al., 2021). The bael fruit is known for its nutritive and medicinal properties, which can be traditionally used to cure many diseases such as cough, fever, and cold (Saini et al., 2022). It is a rich source of carbohydrates, proteins, minerals, vitamins, and various phytochemicals including flavonoids, alkaloids like Angeline, halfordinol, fragrine, aegelenine, and ethyl cinnamate, phenolic acids (ellagic acid, gallic acid, chlorogenic acid, vanillic acid, and protocatechuic acid), tannins (4,7,8‐trimethoxyfuroquinoline), coumarins (xanthotoxol, marmelosin, imperatorin, zanthotoxol, alloimperatorin, isoimperatorin, umbelliferone, scopoletin, methyl ether, psoralen), tocopherols, and carotenes (Hazra et al., 2020; Sharma et al., 2022). Bioactive compounds have revealed potential anticancer, anti‐inflammatory, antiviral, antimicrobial, antidiarrheal, and antipyretic activities of bael fruit (Chandra et al., 2023). For instance, Sharma et al. (2023) studied the antimicrobial activity of mucilage extracted from bael fruit and found the potential of microbial inhibition against *Escherichia coli* and *Staphylococcus aureus*. In addition, polysaccharides are the bael fruit's primary biological active component, which is usually attributed to the many health benefits (Sharma et al., 2022). It is one of the essential biological macromolecules required to maintain the normal functioning of the body.

Structurally, bael fruit polysaccharides are composed of various monomeric units of arabinose, glucose, galacturonic acid, galactose, and rhamnose, which are connected either through (1 → 4) or (1 → 6) glycosidic bonds (Fang et al., 2020; Jindal et al., 2013a, 2013b). They can be converted into monosaccharides by hydrolysis, and the hydrolyzed products include pentose sugar (xylose) and hexose sugar (glucose and galactose) (Ndubuisi et al., 2023). They show excellent functional properties because of hydrogen bonding among the polar and functional groups in their polymer chains, which is important in emulsion formation, gel formation, and capping and stabilization of metal nanoparticles (Castro et al., 2021). Also, bael fruit polysaccharides have a high amount of d‐galactose (54.26%) and glucuronic acid (20.8%). Thus, it results in polysaccharides' good solubility and water‐retaining capacity (Panda et al., 2020). The high galactose and low mannose content improve the solubility of polysaccharides at low temperatures. A high amount of galactose prevents the solid‐state packing of polysaccharides. As a result of the free rotation of the (1 → 6) linkages, the conformational entropy in the solution is increased (Sionkowski et al., 2022). Polysaccharides must have high solubility and water‐retaining capacity for utilization in the various pharmaceutical and food industries to stabilize the emulsion and modify texture and rheological characteristics.

Moreover, they have multiple uses in the food sector, including as coating agents, film‐forming agents, emulsifying agents, stabilizing agents, thickening agents, binding agents, and ingredient replacers like fats (Rahman et al., 2021). Polysaccharides from bael fruit can be obtained by utilizing three most popular techniques including microwave‐assisted extraction (MAE), cold‐water extraction, and hot‐water extraction. The other chemical, physiochemical, and functional properties of polysaccharides depend on the extraction and purification methods (Villacís‐Chiriboga et al., 2020). Over the years, studies have explored the biologically active components and applications of bael fruit polysaccharides. However, there is limited information on the extraction of polysaccharides from bael fruit, providing an opportunity for further investigation and knowledge generation. Hence, this review focused on summarizing the various methods of polysaccharide extraction from bael fruit, including their advantages and disadvantages, characterization, biological activity, and novel food applications.

## 2 EXTRACTION OF POLYSACCHARIDES FROM BAEL FRUIT

Generally, the yield of the polysaccharides depends on the various extraction methods, extraction solvents, and sample characteristics. Several other factors, including temperature, pH, solvent‐to‐liquid ratio, instrumentation technique, and extraction time, are vital in extracting polysaccharides from bael fruit (Hatzakis, 2019). Furthermore, the extraction techniques also affect the polysaccharides' bioactivity and structure. The methods from conventional to advanced have been chosen for the bael fruit polysaccharide extraction based on the intrinsic attributes of the fruit, such as tissue complexity, heat sensibility, and end‐use or application (Kaur et al., 2021; Otoni et al., 2021). Different methods for polysaccharide extraction are categorized into conventional and non‐conventional processes.

Generally, the conventional extraction process is the most common extraction technique to isolate the polysaccharides, mainly including hot water extraction, solvent extraction, and cold‐water extraction (Fang et al., 2020). However, these techniques have some drawbacks, such as requiring a longer time for extraction, high solvent consumption, and low extraction efficiency. To overcome these challenges, new techniques for extraction are being developed as alternatives to traditional methods. The development of new extraction methods aims to enhance the yield and efficiency of polysaccharides (Mena‐García et al., 2019). These methods are fast, more effective, environment‐friendly, safe, and accurate. There are several techniques, including ultrasonic extraction, microwave extraction, enzyme extraction, high‐pressure extraction, supercritical fluid extraction, and pulsed electric field extraction, are used for polysaccharide extraction (Quitério et al., 2022). However, only hot water extraction and MAE techniques have been used to extract polysaccharides from the bael fruit. Presently, there is a great demand for the use of microwave technology in polysaccharide extraction. MAE is an example of a non‐conventional technique that exhibits the appropriate potential to extract polysaccharides (Picot‐Allain et al., 2022). Both conventional and non‐conventional techniques for the extraction of polysaccharides from bael fruit have been described in the following section.

### 2.1 Hot water extraction

**FIGURE 1:**
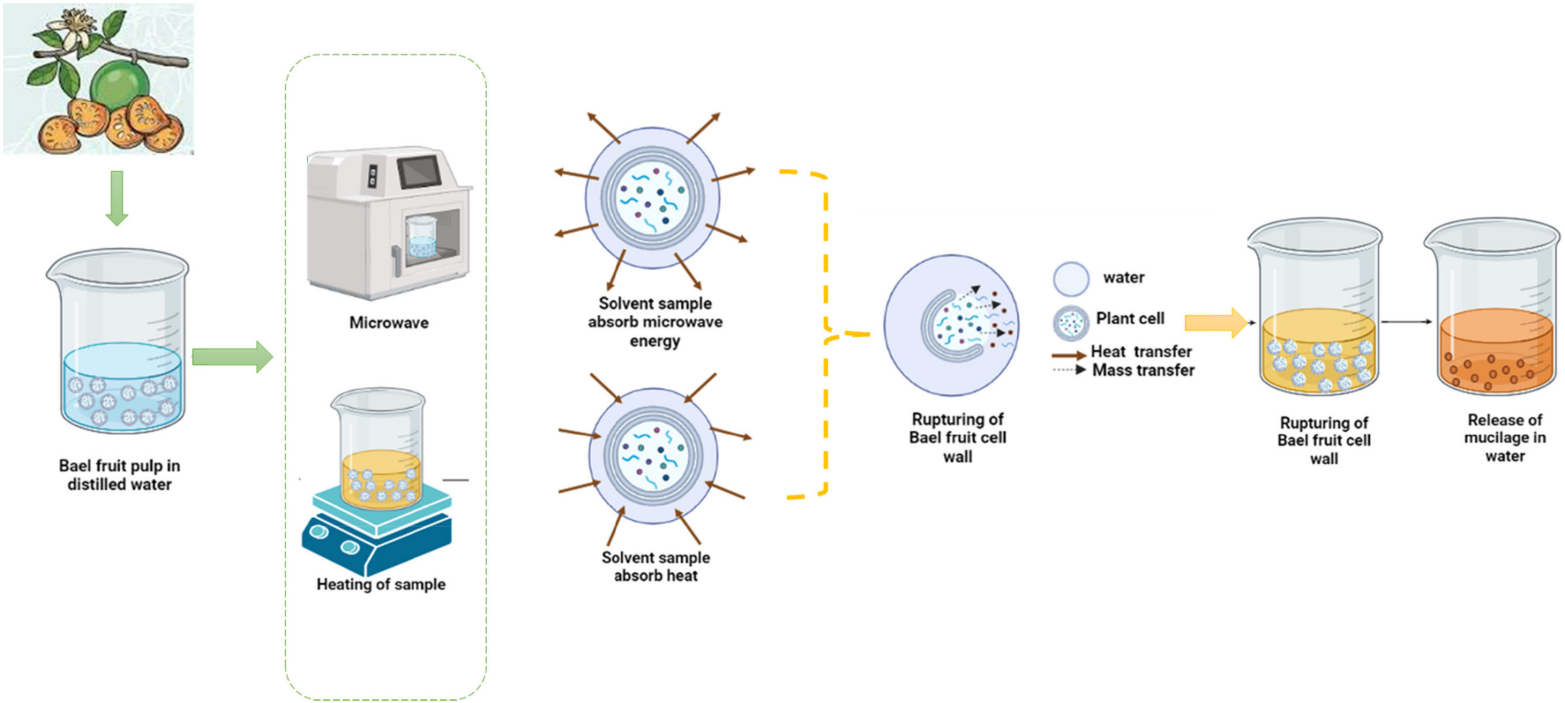
Microwave‐assisted and hot water extraction of polysaccharides from bael fruit.

Most traditional methods rely on the effectiveness of the solvent to extract polysaccharides in addition to the application of agitation and heat (Khadhraoui et al., 2021; Nakhle et al., 2021). Extraction using hot water is the most common and efficient conventional technique, which extracts polysaccharides at a certain temperature and time. This method is widely employed, as it is conventional, easy, and cost‐effective, increasing the polysaccharides' solubility (Gong et al., 2020). The polysaccharides' extraction process can be carried out at a temperature ranging from 90°C to 100°C, and at this temperature range, the water inside the cell matrix is heated up, and evaporation takes place, which creates pressure in high amounts inside the cell wall tissue. Due to that pressure, the cell wall breaks, and the plant matrix becomes more porous, resulting in better penetration of the water, which causes the release of polysaccharides into the water (Figure 1; Drevelegka & Goula, 2020). In this process, mass is transferred from the inside of the sample matrix to the outside of the aqueous matrix, and heat is transferred from the outside of the aqueous matrix to the inside of the sample matrix. This process continues till the polysaccharides emerge into the extracting solvent. However, this method has some limitations, such as requiring high temperature, long extraction time, a high ratio of liquid to solid, high energy, and several extraction steps to get a better yield of polysaccharides (Mena‐García et al., 2019). Patil et al. (2018) extracted the bael fruit polysaccharide using hot water followed by acetone precipitation, which was performed at a temperature of 70–80°C for 30 min. When optimum conditions were applied, the yield of polysaccharides after drying was 14%.

### 2.2 Microwave‐assisted extraction

Due to the several limitations of conventional extraction techniques, innovative, green, and economically feasible techniques are encouraged (Qureshi et al., 2023). These green approaches are of utmost importance due to their inherent environmental benefits, such as minimized use of harmful chemicals and conservation of natural resources. Adopting such techniques in the current era focused on sustainability aligns with global environmental goals. It fosters a responsible and eco‐friendly approach to various industries, contributing to a more sustainable and resilient future (Chen et al., 2019). Compared with conventional methods, non‐conventional require less time and provide better yield with less consumption of organic solvent. Non‐traditional techniques include high‐pressure, supercritical fluid, enzyme, ultrasonic, microwave, and pulsed electric field extraction. Green techniques for the extraction of polysaccharides are novel techniques. It provides energy‐efficient polysaccharide extraction, separation, and purification processes (Ali et al., 2021).

Microwave energy holds great potential as an alternative method for extracting polysaccharides in polar liquids due to the strong penetration power of microwaves. Microwave heating occurs when electromagnetic waves with frequencies between 300 MHz and 300 GHz are produced by microwaves (Zainuddin et al., 2021). For heating household and commercial samples, that is, food, medical, pharmaceutical, and nutraceutical, the frequently applied microwave frequencies are 915 and 2450 MHz. The MAE process's crucial role is its heating, which results in the breakdown of plant tissue and an increased mass transfer rate (Saini et al., 2022). When polar fluid media is used to transfer microwaves, that is, water, the electromagnetic energy is absorbed and changed into heat energy (Costa et al., 2023). During microwave heating, evaporation occurs when microwaves come in contact with moisture inside the cell matrix, and a high amount of pressure is produced inside the cell wall tissue. Microwaves disrupt the cell wall and cause an increase in the plant matrix pores, resulting in better solvent penetration, which facilitates the extraction of polysaccharides into the solvent (Figure 1). Compared with conventional extraction, mass and heat transfer occurs in the same direction, that is, from the inside of the sample matrix to the outside of the aqueous matrix. The result of this mass and heat transfer occurring in the same direction is enhanced solute transfer and the quick collection of more polysaccharides. The MAE method was shown to be more beneficial not just because it has low extraction time, reduced thermal gradients provide higher yield, lowers solvent usage, and requires less amount of energy but also because, in this technique, water is used, which is a polar and environment‐friendly solvent (Mandal et al., 2023). The study evaluated the effectiveness of using MAE to obtain a high yield of polysaccharides, such as mucilage from bael fruit, as summarized in Table 1. The findings demonstrated that MAE was an effective method for polysaccharide extraction, regardless of the type of solvent and conditions used. For example, a study by Kolhe et al. (2014) using MAE at 420 W microwave power for 7 min resulted in a yield of 46%. This is a significant improvement over conventional extraction methods, such as boiling for 1 h, which only resulted in an 8% recovery of polysaccharides.

**TABLE 1:** Different methods used for extraction of polysaccharides from Bael fruit.

Extraction method	Extraction conditions	Yield	References
Direct extraction (Conventional method)	Bael fruit was cut into two halves, and the gummy substance was collected. Drying: 70°C for 24 h (Vacuum drying)		Banu et al. (2020)
Cold water extraction (Conventional method)	Boiling time: 45 min Drying temperature: 50°C (hot air oven)	30%	Jindal et al. (2013a, 2013b), Mirza et al. (2018), Sharma et al. (2021)
Hot water extraction and acetone precipitation (Conventional method)	Soaking of pulp: 24 h Boiling: 1 h After filtration heating temperature: 70–80°C for 30 min Drying temperature: 50°C (hot air oven)	14%	Patil et al. (2018)
Cold water extraction (Conventional method)	pH: 3.5 Heating temperature: 28°C for 24 h	15%	Ibrahim et al. (2016)
Hot water extraction (Conventional method)	Soaking: 24 h Boiling: 1 h Drying: 37°C (incubator)	8%	Kolhe et al. (2014)
Microwave extraction (Non‐conventional Method)	Soaking: 24 h		Kolhe et al. (2014)
	Energy:		
	140 W for 7 min	10%	
	245 W for 7 min	18%	
	420 W for 7 min	46%	
	490 W for 7 min 560 W for 7 min Drying: 37°C (incubator)	(Product degraded)	

## 3 STRUCTURAL CHEMISTRY OF BAEL FRUIT POLYSACCHARIDES

**FIGURE 2:**
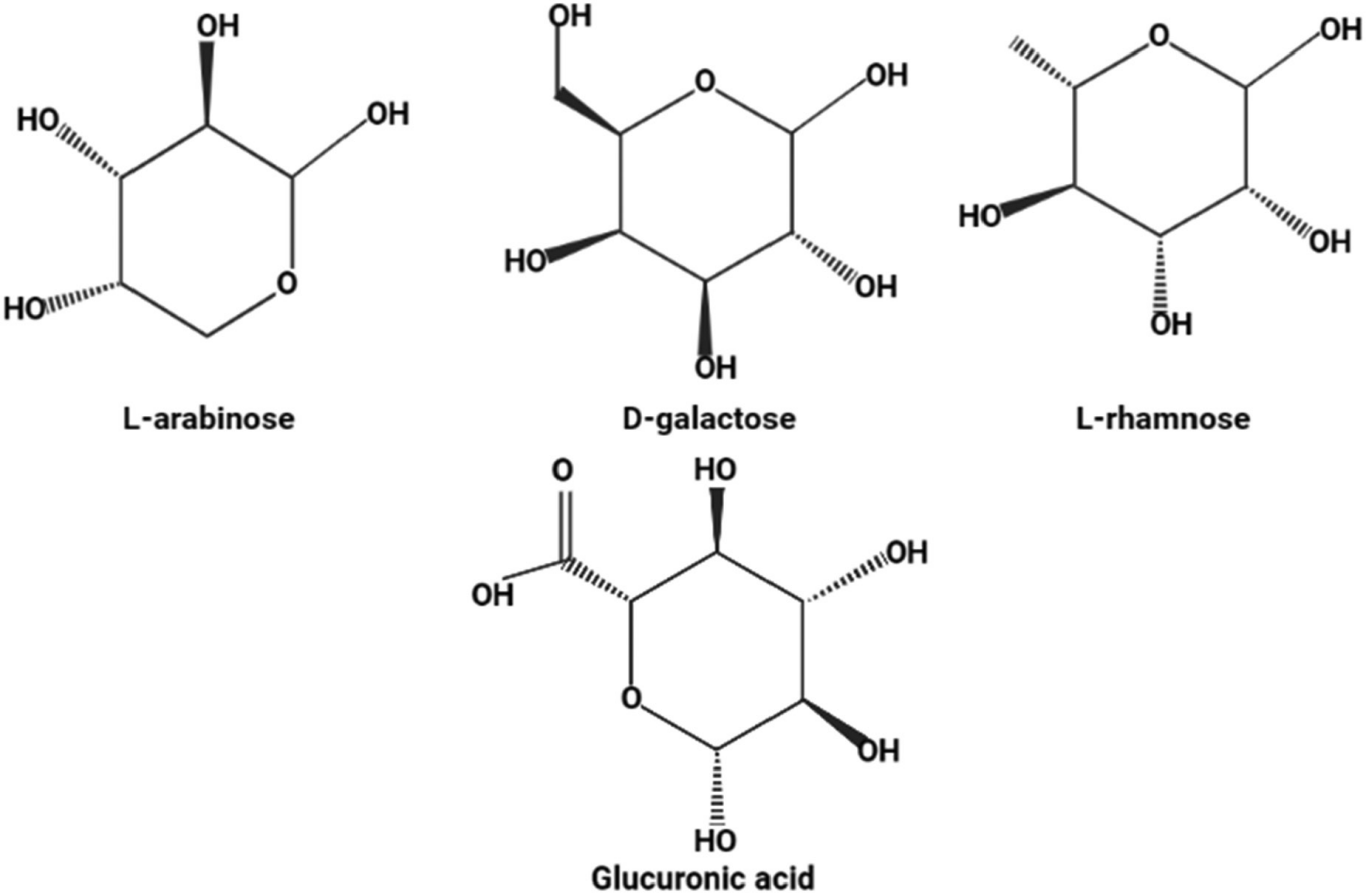
Chemical structure of different mucilage groups of bael fruit.

Bael fruit polysaccharides are long‐chain heteropolysaccharides consisting of various fractions, such as galactose, rhamnose, arabinose, and glucuronic acids, which are present in a molar ratio of 9:3:1:3 (Chintamaneni et al., 2023). The polysaccharides are linked by different glycosidic bonds, which determine their properties and function. On hydrolysis, the polysaccharides can convert into fractions that yield galactose (54.26%), glucuronic acid (20.80%), rhamnose (18.83%), and arabinose (6.10%), as shown in Figure 2 (Jindal et al., 2013a, 2013b). The structure of bael fruit polysaccharides consists of a backbone of d‐galactopyranose with side chains of l‐arabinofuranose, l‐rhamnose, d‐glucuronic acid, and 4‐*O*‐methyl‐d‐glucuronic acid. The methyl group of rhamnose is connected to the C‐4 and C‐6 position of galactose, respectively, which may be due to the two different galactose derivatives.

## 4 PHYSICOCHEMICAL AND TECHNO‐FUNCTIONAL PROPERTIES OF BAEL FRUIT POLYSACCHARIDE

Potential and diverse applications of plant‐derived polysaccharides have been increased in food and pharmaceutical industries due to their techno‐functional properties, biodegradability, less toxicity, biocompatibility with other molecules, and sustainability (Gali et al., 2023). Several researchers revealed various techno‐functional properties, including solubility, water, and oil holding capacity, emulsifying properties, swelling index, and foaming properties of various fruits‐based polysaccharides; however, bael fruit polysaccharides have been explored for their solubility and swelling properties. More functional properties of bael fruit polysaccharides need to be explored for wider industrial applications.

### 4.1 Solubility of polysaccharides due to galactose and glucuronic acid

The solubility of polysaccharides varies greatly, with some being insoluble in water, such as cellulose, some only dissolving in hot water, like starch, and others, such as pullulan and gum arabic, easily dissolving in cold water. The degree of polysaccharide dissolution varies from that of small crystalline molecules, which involves breaking down the crystal structure and releasing the individual ions and atoms. The dissolution of polysaccharides involves constant hydration, in which bonds are converted between polysaccharides and form a bond with water (Singh et al., 2021). Most polysaccharides of a non‐starchy nature are in an amorphous state, and the dissolution process is determined by entropy as molecules attain lower energy conformations. The multiple OH groups are present in the polysaccharides, so they have a strong affinity to bind with water molecules. However, the strong interaction between polysaccharides is due to hydrogen bonding. The polysaccharides require good solubility in water to form emulsion (Li, McClements, et al., 2020). In this context, a study performed by Sharma et al. (2023) on the solubility of bael fruit mucilage revealed that the mucilage has 89.26% solubility, which was due to its highly branched structure of mucilage that provides more sites for the water molecules to bind and form hydrogen bond. The excellent solubility of the polysaccharides is due to the high amount of d‐galactose and glucuronic acid. The high amount of these monomers restricts the solid‐state packing of polysaccharides. In another study, the powder extracted from bael fruit mucilage possessed good solubility in warm water and showed swelling in cold water, whereas it was insoluble in non‐polar solvents. Since mucilage is hydrophilic, it is suitable as a mucoadhesive and binding agent in the pharmaceutical and food industries (Patil et al., 2018). The ability to dissolve in water enables the formation of a viscous and adhesive solution, contributing to the binding property of the mucilage. Overall, the high solubility of the *Aegle marmelos* mucilage makes it suitable to use as a stabilizer and emulsifier in various food applications.

### 4.2 Swelling property of polysaccharides due to galactose

Polysaccharides absorb water and can hold a substantial amount of water. The galactose having more hydrophilic sites (hydroxyl group) is present in polysaccharides, so it can retain water and swell without dissolving. The swelling index of the bael fruit mucilage revealed that it absorbs more than 10 times water by its weight. As a result, including these components in tablet formulations improves the release profile and mucoadhesiveness. The swelling index of the mucilage powder was performed using different pHs, such as distilled water, pH 6.8 phosphate buffer, and pH 1.2 acid buffer, and it was concluded that the swelling of mucilage was pH independent. Hence, incorporating mucilage ensures that it is suitable for use as a mucoadhesive in drug delivery systems (Patil et al., 2018). In conclusion, the galactose‐rich polysaccharides in bael fruit mucilage exhibit remarkable water absorption capacity, as evidenced by their swelling index. The pH‐independent swelling behavior underscores the potential of bael fruit mucilage as a promising and versatile mucoadhesive component for enhancing drug delivery systems.

## 5 CHARACTERIZATION OF BAEL FRUIT POLYSACCHARIDE

Polysaccharides consist of complex structures, and thus, it is necessary to characterize them because they show excellent functional properties and biological activity. Polysaccharides' structural configuration, associated linkage patterns, and functional groups can be determined using various spectroscopic methods (Chen et al., 2019). The functional groups of polysaccharides are evaluated by using Fourier Transform Infrared Spectroscopy (FTIR), and the physical characteristics, such as morphology, thermal decomposition, crystal nature, and particle size, are analyzed by scanning electron microscopy (SEM), thermogravimetric analysis (TGA), X‐ray diffraction (XRD), and zeta potential, respectively (Akhtar et al., 2018; Li, Zhang, et al., 2020).

### 5.1 Fourier transform infrared spectroscopy analysis

**FIGURE 3:**
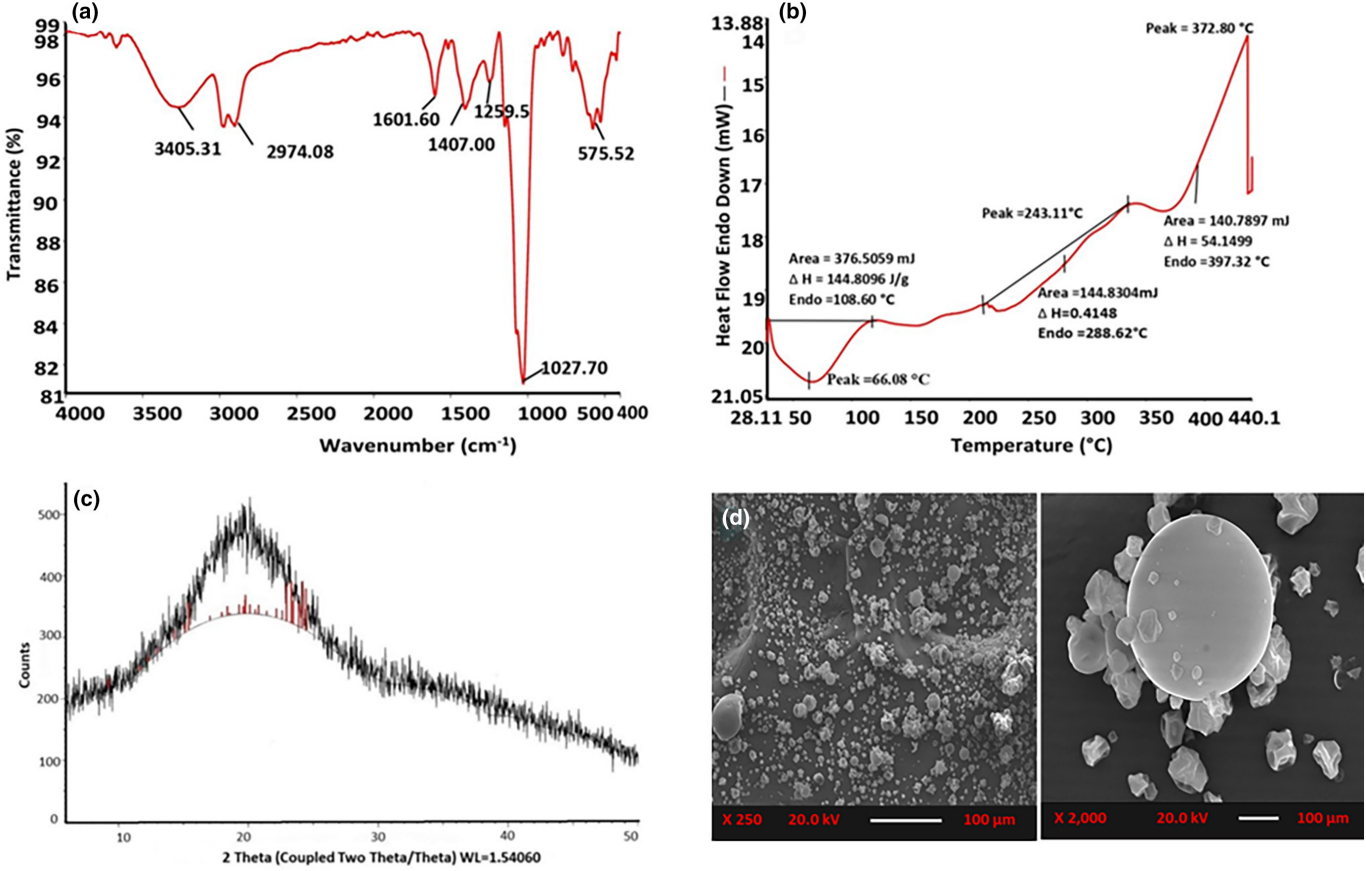
Characterization of bael fruit mucilage. (a) FTIR spectrum, (b) DSC thermogram, (c) XRD diffractogram, and (d) SEM analysis. Figure (a–d) is reproduced with permission (copyright © 2023 Elsevier Ltd., Amsterdam, the Netherlands) from Sharma et al. (2023).

FTIR is used to examine functional groups, composition, conformational changes (impact of temperature, pH, and binding), stability of the structure, and aggregation of polysaccharides. However, it does not define the entire structure of molecules, as it only confirms the configuration of the polysaccharides (Zhang et al., 2021). The fingerprint region of the absorption spectra is considered between 800 and 1650 cm^−1^ because all the major chemical groups of the polysaccharides are present in this region (Dong et al., 2021). The bael fruit polysaccharide mainly comprises OH, C=O, CH_3_, CH, C–O–H, and C–O as major functional groups. It can be inferred from Figure 3a that the bael fruit polysaccharides exhibit a strong absorption band at 3500–3200 cm^−1^, which is mainly due to the hydroxyl group (–OH) stretching, indicating the inter‐ and intrahydrogen bonding among polysaccharide network (Table 2). The asymmetric C–H stretching can be assigned to bands 2926, 2950, 2975, 2922, and 2926.7 cm^−1^, respectively, which indicates the presence of free sugars such as galactose, arabinose, rhamnose, alkane, and aldehyde (Banu et al., 2020). Furthermore, C=O stretching vibrations of the carboxylate group, mainly attributed to uronic acid, can be confirmed at 1750 and 1417 cm^−1^. The spectral fingerprint for galactoprotein due to C=O stretching vibrations of carboxylic acid can be observed between 1076 and 1264 cm^−1^. The presence of the C–O–C ether group can be confirmed by the bending at 1031 cm^−1^, a characteristic of the attachment of two galactose sugar molecules (Patil et al., 2018). Overall, it could be inferred that bael fruit polysaccharides also consist of various carbohydrate and protein fractions.

**TABLE 2:** FT‐IR spectral peaks and functional group of bael fruit polysaccharides.

Wave number (cm^−1^)	Functional group	Reference
3405	OH group	Banu et al. (2020)
2926	Asymmetric C–H stretching indicates the presence of sugar, galactose, arabinose, rhamnose, the presence of alkane CH stretch, and aldehyde CH stretch	
1634 and 1417	Carbonyl stretching vibrations and symmetrical stretch of a carboxylate group due to glucuronic acid	
1076	C–O stretching vibrations of carboxylic acid due to galactoprotein	
3500–3200	OH stretching—hydrogen bonded Amino group masked by broad OH group absorption band	Patil et al. (2018)
2950	C–H stretching indicates the presence of sugar, galactose, arabinose, rhamnose, the presence of alkane CH stretch, and aldehyde CH stretch	
1750–1715	C=O bond—the presence of aldehyde of sugars and the carboxyl group of uronic acid	
2800	C–H stretching—aldehyde group	
1130–1023	C–O, C–C stretching—glycosidic bond	
1304	Carboxylic acid	Mirza et al. (2018)
2926	C–H stretching vibration—indicates the presence of sugar, galactose, arabinose, rhamnose, the presence of alkane CH stretch, and aldehyde CH stretch	
1030	C–O stretching vibration of alcohol due to sugar backbone	
3425	OH stretching inter‐ and intramolecular hydrogen bonding	Srivastava et al. (2015)
2975	CH group stretching—indicates the presence of sugar, galactose, arabinose, rhamnose, the presence of alkane CH stretch, and aldehyde CH stretch	
1644 and 1264	C–O stretch—carboxylic acid due to galactoproteins	
3250–3600	O–H broad peak due to inter‐ and intramolecular hydrogen bonding	Mahammed et al. (2015)
2850	C–H stretching—alkane	
1375	C–H bending of alkane due to sugar backbone	
1031	C–O–C—ether group due to attachment of two galactose sugar	Kolhe et al. (2014)
1668	C=O—aldehyde group	
2922	C–H stretching indicates the presence of sugar, galactose, arabinose, rhamnose, the presence of alkane CH stretch, and aldehyde CH stretch	
3367 and 3385	Primary OH and secondary OH stretching due to inter‐ and intramolecular hydrogen bonding	
3405.2	OH stretching inter‐ and intramolecular hydrogen bonding	Jindal et al. (2013a, 2013b)
1076.4	C–O stretching of carboxylic acid due to galactoproteins	
1634.9	C–O stretching vibration	
2926.7	C–H stretching indicates the presence of sugar, galactose, arabinose, rhamnose, the presence of alkane CH stretch, and aldehyde CH stretch	
1417.3	CH_2_ bending	
1395	OH bending	
1259.5	Carboxylic acid moieties of uronic acid	

### 5.2 Differential scanning calorimetry analysis

The thermal properties of polysaccharides are analyzed by differential scanning calorimetry (DSC), which helps to identify the occurrence of physical and chemical changes. This technique can also examine the glass transition or the melting of bael fruit polysaccharides (Devi et al., 2020; Sabet et al., 2021). The thermal characteristics of bael fruit gum revealed the first endothermic transition at 63.4°C due to dehydration or loss of bound water (Sai Revanth et al., 2019). The second endothermic transition of bael fruit gum was observed at 263.5°C because of the breakage of glycosidic linkage and depolymerization (Figure 3b) (Jindal et al., 2013a, 2013b; Sharma et al., 2023). The exotherm of the DSC thermogram of polysaccharides is due to their decomposition or melting. Another DSC study of bael fruit gum revealed a large peak of endothermic transition (heat absorption) at 105.3°C and released 342.6 J/g of energy (heat of fusion) during the process (Srivastava et al., 2015). The thermostability of the polysaccharides is directly related to the thermal transition temperature. Those with higher thermal transition temperatures are more stable and have more applications in food that require extreme temperatures (Hamdani et al., 2019). Overall, it can be revealed that bael fruit polysaccharides could be an excellent additive to formulate food products that require high‐temperature processing.

### 5.3 X‐ray diffraction

The powdered polysaccharides are examined for their crystal structure and orientation using a non‐destructive technique, X‐ray diffraction. This technique also provides information on various other structures, such as strain, crystal defects, and size and phases (Figure 3c). This technique has been extensively increased in the food industries due to its wide range of applications (da Costa Pinto et al., 2023; Olakanmi et al., 2023). The intensity of diffraction peaks is higher in crystalline polymers. In contrast, due to the breakage of hydrogen bonds between the chains of an amorphous polymer, there is a reduction in XRD peak intensity (Srivastava et al., 2015). Overall, the bael fruit polysaccharide is an amorphous material with no sharp peaks. The absence of sharp peaks in the diffraction revealed the amorphous nature of the polysaccharides.

### 5.4 Scanning electron microscopy

Scanning electron microscopy is a valuable approach for investigating polysaccharides' microstructure and surface morphology. It uses a low‐energy beam of electrons to scan the surfaces of samples. SEM is preferred for particle size analysis because of its high resolution (Yan et al., 2021; Yang et al., 2020). Bael fruit polysaccharides' surface morphology and microstructure are crucial as they determine the material's physical characteristics. These attributes influence the polysaccharides' performance in applications such as film formation, where a well‐defined microstructure enhances mechanical strength and barrier properties. In a study, the surface morphology of synthesized carboxylated bael fruit gum as beads using metformin was characterized by SEM, in which the mucoadhesive property was maximized. The scanning electron microscopy analysis results showed that the metformin‐loaded bael fruit gum (BFG) beads had a smooth surface and spherical shape. In contrast, the metformin‐loaded CBFG (carboxylated bael fruit gum) beads had an irregular shape with a rough and porous surface (Srivastava et al., 2015). Another study by Sharma et al. (2023) investigated the scanning electron microscopy of bael fruit mucilage and found that the uniform spherical‐shaped particles have concavities in their internal structure (Figure 3d). Overall, the structure of the bael fruit mucilage revealed its potential in various food applications such as stabilizer, emulsifier, and encapsulant.

## 6 APPLICATION OF BAEL FRUIT POLYSACCHARIDES

### 6.1 Emulsifying agent

**FIGURE 4:**
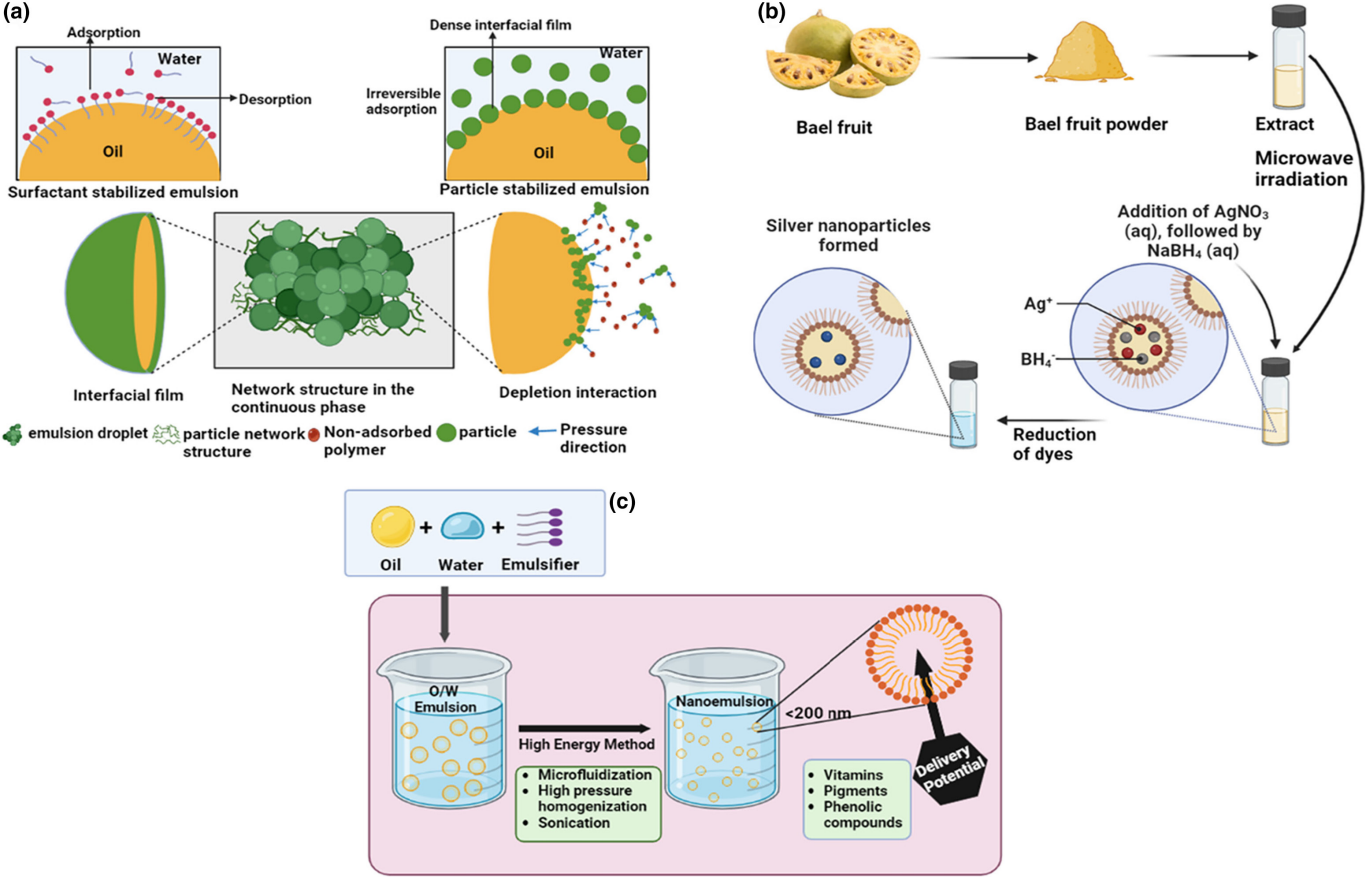
Functional properties of polysaccharides and its mechanism. (a) Emulsifying agent, (b) reduction of dyes, and (c) nanocarrier.

Because of its extensive applications, the polysaccharide emulsification property is important in several food industries. An emulsion is a homogenous mixture of two liquids, which are typically immiscible, where one liquid is dispersed as droplets within the other (Yang et al., 2020). Various types of emulsions are utilized in the food, cosmetic, and pharmaceutical industries. However, the emulsions are thermodynamically unstable, which can lead to the separation of phases due to creaming, ripening, and coalescence. Emulsifying agents and stabilizers formulate the stable emulsion (Costa et al., 2019). Emulsifiers help in the formation of fine emulsions by decreasing the interfacial tension and preventing the coagulation of emulsion. In contrast, stabilizers are hydrophilic polymers like proteins and polysaccharides that change the rheological properties and stabilize the emulsion (Figure 4a). The polysaccharides increase the viscosity of the constant phase in the colloid solutions, which delays the movement of the particles and droplets, leading to an increase in the stability of the emulsion. The most common synthetic emulsifiers are mono and diglycerides, sorbitol, propylene glycol esters, polyglycerol esters, polysorbates, and phosphates (Marhamati et al., 2021). Polysaccharides act as natural emulsifiers, which are in more demand because of the harmful effects of synthetic emulsifiers. The natural emulsifiers include lecithin, guar gum, gellan gum, gum arabic, xanthan gum, and carrageenan gums.

The emulsification property of bael fruit gum has been compared with gum arabic, and it was found to have a superior ability to form an emulsion and maintain stability (98.2%) (Jindal et al., 2013a, 2013b). Highly branched structure and high molecular weight are responsible for peach gum, xanthan gum, and gum arabic emulsification properties, respectively (Wei et al., 2019). The bael fruit gum comprises d‐galactose, d‐glucuronic acid, l‐rhamnose, and l‐arabinose. Hence, the highly branched structure may be responsible for improved emulsification properties.

### 6.2 Binding agent

Polysaccharides act as binders because they impart cohesiveness to the powder mass and are sticky, holding the polysaccharide powder together and forming granules. To overcome synthetic binding agents, various polysaccharides such as gums and mucilage are used as they exhibit the binding properties. Gums and mucilage have been used in tablet formation as binding agents to sustain drug release. The ability of bael fruit gum to act as a binding agent in tablet drug formulations was studied, and concluded that the friability value decreases and the hardness of the tablet increases with increases in the concentration of bael fruit gum (Kharwade et al., 2011). In another study, a tablet was formulated by substituting synthetic polymeric binder with bael fruit gum and evaluated for uniformity, hardness, friability, and disintegration time. It has been observed that as the concentration of bael fruit gum increases, the hardness also increases, whereas there is a decrease in the friability values of tablets. In addition, the study found that tablets made with bael fruit gum took longer to disintegrate than those made with starch. These results indicate that bael fruit gum is a more effective binding agent than other polymers and can be a cost‐effective alternative (Kolhe et al., 2014).

### 6.3 Reduction of hazardous dyes

The contamination of water, which can harm both the environment and human health, is caused by the use of dyes in different industries such as textile, paper, and plastic industries. While there are methods to reduce or remove dyes, they are often costly and pose challenges in disposing of treated materials (Bushra et al., 2021). An alternative is to use naturally occurring polysaccharides, such as gums, mucilage, and chitin, as complexing and chelating agents to remove dyes from effluent. These substances have a high number of chelation sites, due to which they can absorb dyes and heavy metals from a solution by binding to them (El‐Gaayda et al., 2021). An environment‐friendly method (microwave‐induced) for synthesizing silver nanoparticles, which utilizes bael fruit gum as both a stabilizer and reductant, has been studied. This method has the potential for use in reducing organic dyes. The biomolecules in bael fruit gum effectively reduced silver ions and stabilized the resulting silver nanoparticles, as confirmed by FTIR spectroscopy. Additionally, the reduction of dyes such as 4‐Nitrophenol, Rhodamine B, and Methylene Blue was catalyzed using silver nanoparticles in the presence of NaBH_4_ (Figure 4b) (Banu et al., 2020).

### 6.4 Application of polysaccharides in film formation

Polysaccharides exhibit desirable food utilization attributes, including being non‐toxic, having good biocompatibility, biodegradability, ease of processing, and moldability. Consequently, they can create films, making them especially valuable in food preservation. Films are widely used as a coating to prolong the shelf life of perishable fruits and vegetables. Polysaccharides have extensive applications in developing edible coatings; one example is using a film made from a blend of bael fruit gum and chitosan to improve film‐forming properties (Jana & Jana, 2020). The film containing equal parts chitosan and bael fruit gum showed the lowest swelling index (2.3 ± 0.11). The minimum value was likely due to the optimal interaction between chitosan and bael fruit gum, leading to a highly cross‐linked interpenetrating network. Furthermore, FTIR and DSC analysis results of the films indicated that the COO^−^ groups of the BFG interacted with the NH^3+^ of chitosan, resulting in a lipophilic film with minimal functional groups that can interact with water. The film's hydrophilic or lipophilic character was determined by observing the contact angle created by the buffer solution at pH 1.2, 6.8, or 7.4 with the film surface. An increase in the contact angle indicates a less hydrophilic (more lipophilic) property of the film (Tian et al., 2023). The work of adhesion represents the force necessary to separate the buffer and film. The stronger the bonding between the buffer and film surface, the higher the work of adhesion. Regardless of the pH of the buffer, the film had the highest contact angle, suggesting its lipophilic nature and a lower Swelling index. The film also had almost no zeta potential, and when examined under SEM, its surface was smooth after being exposed to alkaline and acidic pH. It has been found that the film can be used in processed food and to modify drug release (Jindal et al., 2013a, 2013b).

### 6.5 Application of polysaccharide as a nanocomposite

Polysaccharides are hydrophilic, so they have excellent potential to stabilize the emulsion. It is in high demand because of its unique properties, like biological characteristics, biocompatibility, and physicochemical properties, which make it suitable for broad‐spectrum utilization. Nano‐sized particles exhibit more stability and have many potential applications, including controlled drug release, film production, dye removal, and encapsulation. Besides that, they have low immunogenicity and are biodegradable. Polysaccharides are composed of functional groups such as hydroxyl, amino, and carboxyl groups, which provide interaction with biological tissues. The polysaccharides‐based nanocarrier is highly valued and utilized for drug delivery in the pharmaceutical and food industries (Samrot et al., 2020). The bael fruit gum is a nanocomposite and chitosan for bone tissue engineering. The carboxylic acid moieties of bael fruit gum enhance mechanical stability by creating an interfacial interaction among the inorganic and organic phases (Figure 4c) (Mirza et al., 2018). Different applications of bael fruit polysaccharides are summarized in Table 3.

**TABLE 3:** Application of *Aegle marmelos* fruit polysaccharides.

Properties	Application	Reference
Binding agent	Bael gum‐based bio adhesive used for multilayer packaging films	Sharma et al. (2021)
Film‐forming agent	Bael fruit gum is co‐processed with chitosan to improve its film‐forming property	Jindal et al. (2013a, 2013b)
Binding agent	Used as a matrix system for sustaining drug release	Kharwade et al. (2011)
Mucoadhesive material	Mucilage is hydrophilic, making it suitable for use as a binding agent in pharmaceuticals. Incorporation of mucilage in tablet formulation would control release profile and mucoadhesiveness	Patil et al. (2018)
Reducing agent	Used as a catalyst in the reduction of organic dyes	Banu et al. (2020)
As nanocarrier	Incorporated bael fruit gum in chitosan/nano‐hydroxyapatite nanocomposite forming a ternary bioactive nanohybrid for bone tissue engineering	Mirza et al. (2018)

## 7 BIOLOGICAL ACTIVITIES OF BAEL FRUIT POLYSACCHARIDE

Bael is a traditional medicinal plant that has broad nutritional and health benefits. Its various parts are rich in bioactive compounds like flavonoids, phenolic acids, alkaloids, terpenoids, carbohydrates, tannins, and coumarins and these compounds possess several therapeutic properties including antibacterial, antioxidant, antifertility, antidiarrheal, antiproliferative, antidiabetic, antiviral, analgesic, cardioprotective, gastroprotective, anti‐ulcerative colitis, anti‐arthritis, immunomodulatory, lipid peroxidation inhibition, hepatoprotective, and radioprotective (Sharma et al., 2024). The bioactive components in the bael fruit effectively treat several diseases. It has been used since ancient times to treat intestinal ulcers, chronic constipation, dysentery, urinogenital disorder, chronic gastrointestinal disorder, and lower blood sugar levels (Venthodika et al., 2021). Various factors can affect the bioactivity of polysaccharides, including their molecular weight, uronic acid content, primary structure, the composition of monosaccharides, degree of branching, number of branches, types of glycosidic bond, water solubility, polymer charge, and other factors.

### 7.1 Anticancerous activity

Cancer is a major health problem, so it is important to know the mechanism of the uncontrolled division of cells. The antitumor effects of polysaccharides isolated from natural resources, such as plants, are due to their ability to inhibit tumor growth, induce apoptosis, and boost immune function without causing any side effects (Kiddane & Kim, 2021). The physicochemical properties of bael fruit polysaccharides make them favorable for interacting and binding to surface receptors on tumor cells, which can induce apoptosis in the tumor cells. Polysaccharide‐rich bael extract promotes the body's immune system's ability to fight cancer. Rhamnose, galacturonic acid, galactose, arabinose, and rhamnogalacturonan backbone showed possible anticancer action (Dammak et al., 2019). Cell division was reduced when polysaccharide‐rich bael extract was applied to mice with xenografts of HEp‐2 alveolar epithelial and HCT‐116 colon cancer. Malignant cells in the G1 phase of the cell cycle were seen to induce apoptosis simultaneously. An increase in caspase‐3 and caspase‐9 inhibits the cycle during the G1 phase, hence showing antitumor activity (Sarkar et al., 2020).

### 7.2 Antioxidant activity

The significant increase in free radical formation within the body system results in an elevation of oxidative stress, which can damage T cells, reduce immune function, and contribute to aging. The arabinogalactan protein has a main galactopyranosyl chain with β‐1,3‐linkages and side chains containing galactose and arabinose residues attached to the O‐6 position (Maity et al., 2021). The arabinogalactan‐rich fraction was evaluated for its in vitro antioxidant activity using various methods, including radical DPPH scavenging, hydroxyl radical scavenging, radical anion superoxide scavenging, and the reducing power method (Jindal, Rana, et al., 2013). The monosaccharide composition also plays a major role in the antioxidant mechanism of polysaccharides. Bael fruit extract is rich in rhamnose and arabinose, which prevents oxidative damage to lipids. Likewise, aloe vera polysaccharides have a greater concentration of these sugars, exhibiting antioxidant properties by inhibiting the generation of reactive oxygen species (ROS). The high amount of uronic acid also showed maximum antioxidant activity. The molecular size of polysaccharides also plays an important role in their antioxidant properties (Dammak et al., 2019).

### 7.3 Antidiabetic activity

The bael also protects against pathophysiological diseases, including inflammation and oxidative stress. Polysaccharide‐rich fruit juice decreases the effects of oxidative stress by decreasing hydroperoxide levels, lipid peroxidation, and conjugated diene while increasing catalase, glutathione, and hyperoxide dismutase levels in the serum and liver (Sarkar et al., 2020). Research has shown that bael fruit juice is more effective than the antidiabetic medication glibenclamide when consumed at 250 mg/kg. Bael fruit contains l‐arabinose sugar, which has selective inhibitory effects on sucrase activity in the intestines. This results in the suppression of increased blood glucose levels after sucrose ingestion. Additionally, l‐arabinose helps regulate blood glucose levels, leading to a reduction in obesity. In experimental diabetic rats, bael fruit is a viable substitute for reducing blood glucose levels and glycosylated hemoglobin, which causes a rise in liver glycogen. In a different research, rats with normal fasting blood glucose (FBG), normal blood sugar levels, and slightly diabetic (FBG‐120–250 mg/dL) rats received oral dosages of an aqueous extract of bael seeds in the ranges of 100, 250, and 500 mg/kg. The results showed a 25.49% drop in total cholesterol (TC), a 33.43% increase in high‐density lipoprotein (HDL), a 53.97% decrease in low‐density lipoprotein (LDL), and a 45.77% increase in triglycerides (TC). The findings clearly show antidiabetic effects (Sarkar et al., 2020).

## 8 CONCLUSION

This paper provides an overview of bael fruit polysaccharides, a bioactive compound with medicinal properties and health benefits. These polysaccharides are considered valuable for developing new drugs or improving drug efficacy due to their numerous bioactivities and therapeutic effects. Despite advancements in extraction techniques, improvement is still required. The study showed that microwave‐assisted extraction had a higher yield and shorter extraction time than other methods, but each method had different impacts on the polysaccharides. Therefore, there is a need to optimize existing methods or develop new ones for extracting bael fruit polysaccharides. The extracted polysaccharide can be used as a food additive for improving food quality and is thus used on a commercial scale that improves food quality and attributes qualities to functional foods. The study's main limitation was variability in the chemical composition of bael fruit polysaccharides due to factors like geographical location and seasonal variations needing to be thoroughly investigated. Further research is also needed to explore its potential applications in pharmacology and food fully. To fully realize the potential of bael fruit polysaccharides, a focus on their isolation, advanced research, and effective use in various fields such as food and pharmaceuticals is necessary.

## AUTHOR CONTRIBUTIONS

**Madhu Sharma:** Conceptualization (equal); data curation (equal); formal analysis (equal); funding acquisition (equal); investigation (equal); resources (equal); software (equal); supervision (equal); validation (equal); visualization (equal); writing – original draft (equal). **Aarti Bains:** Conceptualization (equal); formal analysis (equal); funding acquisition (equal); investigation (equal); methodology (equal); resources (equal); validation (equal); writing – review and editing (equal). **Gulden Goksen:** Conceptualization (equal); formal analysis (equal); funding acquisition (equal); methodology (equal); project administration (equal); resources (equal); supervision (equal); visualization (equal); writing – original draft (equal); writing – review and editing (equal). **Kandi Sridhar:** Conceptualization (equal); data curation (equal); formal analysis (equal); investigation (equal); software (equal); supervision (equal); visualization (equal). **Minaxi Sharma:** Conceptualization (equal); data curation (equal); funding acquisition (equal); investigation (equal); methodology (equal); software (equal); validation (equal); writing – original draft (equal). **Amin Mousavi Khaneghah:** Conceptualization (equal); funding acquisition (equal); investigation (equal); project administration (equal); supervision (equal); validation (equal); writing – original draft (equal); writing – review and editing (equal). **Prince Chawla:** Funding acquisition (equal); investigation (equal); methodology (equal); project administration (equal); validation (equal); visualization (equal); writing – review and editing (equal).

## CONFLICT OF INTEREST STATEMENT

The authors declare that the research was conducted without any commercial or financial relationships that could be construed as a potential conflict of interest.

## Data Availability

Data will be available based on the reasonable request.
